# Development of a recursive partitioning analysis for prediction of radiation necrosis following single-fraction stereotactic radiosurgery for intact brain metastases

**DOI:** 10.1007/s11060-025-05062-5

**Published:** 2025-05-13

**Authors:** Anirudh Bommireddy, Zachary S. Mayo, Chandana A. Reddy, Cole Billena, Erik M. Davies, Robin W. Davis, Erin S. Murphy, John H. Suh, Ehsan H. Balagamwala, Timothy A. Chan, Jennifer S. Yu, Gene H. Barnett, Lilyana Angelov, Alireza M. Mohammadi, Glen H. J. Stevens, Matthew Grabowski, David M. Peereboom, Samuel T. Chao

**Affiliations:** 1 Department of Radiation Oncology, Cleveland Clinic, Cleveland, OH USA; 2 Tennessee Oncology, Nashville, TN USA; 3 Deparment of Neurosurgery, Cleveland Clinic, Cleveland, OH USA; 4 Department of Neuro-Oncology, Cleveland Clinic, Cleveland, OH USA; 5 Department of Radiation Oncology, Taussig Cancer Institute, Cleveland Clinic, Cleveland, OH 44195 USA

**Keywords:** Brain metastasis, Stereotactic radiosurgery, SRS, Radiation necrosis, Recursive partitioning analysis

## Abstract

**Purpose/objective:**

Radiation necrosis (RN) is a potential complication after stereotactic radiosurgery (SRS) for brain metastases. This study develops a recursive partitioning analysis (RPA) to identify patients at risk for RN following SRS.

**Methods:**

Patients who underwent single-fraction SRS for intact brain metastases at a single institution from 2017 to 2021 were identified. Cox regression identified factors associated with RN, and variables with *p* < 0.1 were included in the RPA. Patients with staged SRS, incomplete records, or less than 3 months of follow-up were excluded.

**Results:**

The study included 170 patients with 919 lesions, with median follow-up of 9 months. Primary disease sites were non-small cell lung cancer (NSCLC, 49%), breast cancer (12%), melanoma (11%), renal cancer (6%), and others (22%). Median prescription dose was 24 Gy, and median maximum lesion dimension (MLD) was 0.7 cm. RN occurred in 110 (12.2%) lesions, of which 32 (3.5%) were symptomatic, at median of 4.9 months after SRS. Variables for RPA included primary disease site, tumor location, MLD, prior SRS, number of SRS targets, dosimetry, prior hemorrhage, and concurrent systemic therapy. RPA identified four groups: Group 1 (MLD ≤ 0.8 cm, non-breast/NSCLC/renal), Group 2 (MLD ≤ 0.8 cm, breast/NSCLC/renal), Group 3 (MLD > 0.8 cm, no post-SRS hemorrhage), and Group 4 (MLD > 0.8 cm, post-SRS hemorrhage). Two-year RN free survival was 99% (Group 1), 89% (Group 2), 70% (Group 3), and 52% (Group 4).

**Conclusion:**

This is the first RPA model for RN after single-fraction SRS, which may aid in risk assessment and distinguishing RN from tumor progression.

## Introduction

The incidence of brain metastases continues to increase as patients are surviving longer from metastatic cancer, with estimates as high as 20–40% of adults with cancer [1]. Stereotactic radiosurgery (SRS) is a standard of care treatment for brain metastases, with the American Society for Radiation Oncology (ASTRO) recommending strong support for SRS in patients with ≤ 4 brain metastases, with conditional recommendations for SRS in those with 5–10 lesions, and another study suggesting benefit for up to 15 lesions [1–3]. Single-fraction SRS is commonly utilized for smaller lesions, with standard prescription doses up to 24 Gy [4].

Though SRS provides excellent local control for brain metastases, cerebral radiation necrosis (RN) is a late complication of treatment that may occur months to years later in up to 20% of patients [1]. Differentiating RN from tumor progression is one of the primary challenges in the post-SRS setting, as both RN and tumor recurrence can appear as enhancing lesions with surrounding edema on magnetic resonance imaging (MRI) [5]. Several tools, including perfusion MRI, 18 F-fluorodeoxyglucose (FDG) positron emission tomography (PET), and MR spectroscopy can aid in identifying RN, but the diagnosis remains a challenge [6]. Symptoms of RN are often managed medically, whereas tumor recurrence may require surgical intervention or further SRS.

Several risk factors for RN have been reported, including radiation dose, lesion volume, prior radiation treatment, primary tumor histology, location, and concurrent therapies [7–15]. However, to our knowledge, there are no recursive partitioning analysis (RPA) models to identify patients at highest risk for RN. We therefore sought to develop a predictive tool to identify patients at highest risk for the development of RN following single-fraction SRS.

## Methods and materials

### Patients and data collection

Patients treated with single-fraction SRS for brain metastases between 2017 and 2021 were identified in an IRB approved, single-institution database. All patients were treated with the Gamma Knife Radiosurgery Icon system (Elekta, Stockholm, Sweden). Patient, treatment, and oncologic specific variables were extracted from our institutional brain metastases Research Electronic Data Capture (REDCaP) database.

Patients treated with definitive SRS for intact brain metastases were included in the analysis. Target dose and volumes were at the discretion of the treating physicians based on clinical presentation. Current institutional practice is to treat patients with tumors < 2 cm with single-fraction SRS and patients with tumors ≥ 2 cm with a staged approach; importantly, no planning target volume (PTV) margin is used. Patients were excluded from this analysis if they were treated with staged SRS, had less than 3 months of radiographic follow-up, or had incomplete or unavailable treatment records.

The primary endpoint of this analysis was development of RN after SRS. Patients with concern for RN vs. tumor progression were discussed in a multidisciplinary setting and a final diagnosis of RN was made based on pathologic or radiographic findings. If a diagnosis of RN was uncertain on conventional MRI, advanced imaging techniques such as perfusion MRI or FDG PET were utilized. Patients with a definitive or suspected diagnosis of RN were included as events in the RPA.

### Variables

Several variables were selected for consideration of inclusion into the RPA: age, gender, primary tumor pathology, intracranial location (supratentorial vs. infratentorial vs. brainstem), concurrent systemic therapy, pre- or post-SRS hemorrhage, prior SRS to other lesions, prior whole brain radiation therapy (WBRT), salvage SRS of a previously treated lesion, history of RN at a different treatment site, number of SRS targets, prescription dose, maximum dose, maximum lesion dimension (MLD), target volume, prescription isodose line, heterogeneity index, conformality index, gradient index, lesion V10, and lesion V12. Variables that were dependent on each other (e.g. target volume and lesion dimension) were only entered into the RPA as a single variable. Receipt of concurrent targeted therapy, chemotherapy, and immunotherapy were defined as treatment within 2 weeks of SRS. Lesion V10 and V12 were defined as the volume of normal brain that received at least 10 Gy and 12 Gy, respectively.

### Statistical analysis

Descriptive statistics were used to report patient and tumor characteristics. Cox proportional hazards regression analysis was performed to identify factors associated with RN, and variables with a *p* < 0.1 on univariate analysis from the Cox regression analysis were included in the RPA. RPA was performed to categorize patients into distinct risk groups for development of RN. Descriptive statistics and Cox proportional hazards regression analysis were done using SAS v 9.4 (SAS Institute, Cary, NC). R v4.2.2 (R Foundation for Statistical Computing, Vienna, Austria), with the packages survival [v3.5-0; Therneau T (2023)] and rpart [v 4.1.1; Therneau T, Atkinson B (2022)], was used for the development of the RPA model.

## Results

### Patient, lesion, and treatment characteristics

With a median follow-up of 9 months (range: 3–61), there were 170 patients with 919 brain metastases (Table 1). The median age was 64 years (range: 22–91), and 88 patients with 523 (57%) lesions were female. The median Karnofsky Performance Status (KPS) was 80 (range: 40–100). Of the 919 lesions, the primary disease sites included 453 (49%) non-small cell lung cancer (NSCLC), 108 (12%) breast cancer, 102 (11%) melanoma, 55 (6%) renal cancer, and 201 (22%) other. The majority (*n* = 734, 80%) of brain metastases were supratentorial. Ninety-seven patients with 284 (31%) lesions had prior SRS to other lesions, and 15 patients with 60 (7%) lesions received prior WBRT. Seven (1%) lesions had been treated with prior SRS to the same location. Pre-SRS hemorrhage was present in 75 (8%) lesions, and post-SRS hemorrhage was identified in 164 (18%) lesions.

**Table 1:** Patient, lesion, and treatment characteristics

Characteristic		Overall *n* = 919
Age (years)	Median (Range)	64 (22–91)
KPS	Median (Range)	80 (40–100)
Gender	Female	523 (57%)
	Male	396 (43%)
Primary Site	NSCLC	453 (49%)
	Breast	108 (12%)
	Melanoma	102 (11%)
	Renal	55 (6%)
	Other	201 (22%)
Location	Supratentorial	734 (80%)
	Cerebellum	169 (18%)
	Brainstem	16 (2%)
Targeted Therapy	No	728 (79%)
	Yes	191 (21%)
Immunotherapy	No	723 (79%)
	Yes	196 (21%)
Chemotherapy	No	783 (85%)
	Yes	136 (15%)
Pre-SRS Hemorrhage	No	844 (92%)
	Yes	75 (8%)
Post-SRS Hemorrhage	No	755 (82%)
	Yes	164 (18%)
Prior SRS to Other Lesions	No	635 (69%)
	Yes	284 (31%)
Prior WBRT	No	859 (93%)
	Yes	60 (7%)
Salvage SRS to Same Lesion	No	912 (99%)
	Yes	7 (1%)
Prior RN at Other Lesions	No	852 (93%)
	Yes	67 (7%)
Number of SRS Targets	Median (Range)	4 (1–21)
Prescription Dose (Gy)	Median (Range)	24 (15–24)
Maximum Dose (Gy)	Median (IQR)	28.9 (26.7–36.4)
Maximum Lesion Dimension (cm)	Median (IQR)	0.7 (0.5–1.1)
Target Volume (cc)	Median (IQR)	0.1 (0.03–0.5)
Prescription Isodose Line	< 70%	312 (34%)
	≥ 70%	607 (66%)
Heterogeneity Index	Median (IQR)	1.2 (1.1–1.6)
Conformality Index	Median (IQR)	1.9 (1.7–2.2)
Gradient Index	Median (IQR)	4.6 (3.3–7.3)
V10 (cc)	Median (IQR)	1.2 (0.7–5.3)
V12 (cc)	Median (IQR)	0.9 (0.6–4.2)

All patients were treated with single-fraction SRS. The median number of lesions treated was 4 (range: 1–21), and the median prescription dose was 24 Gy (range: 15–24). The median maximum dose was 28.9 Gy (IQR: 26.7–36.4). The median MLD was 0.7 cm (IQR: 0.5–1.1), and median target volume was 0.1 cc (IQR: 0.03–0.5). The median prescription isodose line was 82% (IQR: 62–90), median heterogeneity index was 1.2 (IQR: 1.1–1.6), median conformality index was 1.9 (IQR: 1.7–2.2), and median gradient index was 4.6 (IQR: 3.3–7.3). The median V10 was 1.2 cc (IQR: 0.7–5.3) and median V12 was 0.9 cc (IQR: 0.6–4.2). Within two weeks of treatment with SRS, 32 patients with 191 (21%) lesions received targeted therapy, 36 patients with 196 (21%) lesions received immunotherapy, and 24 patients with 136 (15%) lesions received chemotherapy. RN was diagnosed in 110 (12%) lesions, of which 32 (3.5%) were symptomatic. The median time to RN after SRS was 4.9 months (range: 1.3–37.5). For patients that had imaging or clinical findings concerning for RN, diagnosis was confirmed with perfusion MRI for 76 (69.1%) lesions, response to empiric RN treatment for 24 (21.8%) lesions, surgical pathology for 8 (7.3%) lesions, and FDG PET for 2 (1.8%) lesions.

### Cox proportional hazards regression analysis

Several lesion, treatment, and SRS specific characteristics were predictive for the development of radiation necrosis on univariate analysis (Table 2). Larger lesion size (*p* < 0.0001), larger target volume (*p* < 0.0001), primary disease site (*p* = 0.006), lesion location (*p* = 0.089), number of SRS targets (*p* = 0.003), prior SRS to other lesions (*p* = 0.013), presence of hemorrhage prior to SRS (*p* = 0.055), and development of post-SRS hemorrhage (*p* < 0.0001) were significantly associated with development of radiation necrosis.

**Table 2:** Cox proportional hazards regression for RN

Characteristic	HR (95% CI)	*p*-value
Age (years)	1.00 (0.98–1.02)	0.809
Gender (Female vs. Male)	0.83(0.57–1.21)	0.342
Primary Site		
Breast vs. Renal	0.68 (0.30–1.58)	0.372
Melanoma vs. Renal	0.16 (0.04–0.59)	0.006
NSCLC vs. Renal	0.65 (0.32–1.32)	0.235
Other vs. Renal	0.65 (0.30–1.40)	0.272
Location		
Brainstem vs. Supratentorial	2.39 (0.88–6.52)	0.089
Cerebellum vs. Supratentorial	1.04 (0.65–1.68)	0.860
Targeted Therapy (No vs. Yes)	0.64 (0.42–0.97)	0.034
Immunotherapy (No vs. Yes)	1.72 (0.98–3.02)	0.057
Chemotherapy (No vs. Yes)	3.02 (1.33–6.88)	0.009
Pre-SRS Hemorrhage (No vs. Yes)	0.57 (0.32–1.01)	0.055
Post-SRS Hemorrhage (No vs. Yes)	0.38 (0.26–0.56)	< 0.0001
Prior SRS to Other Lesions (No vs. Yes)	1.81 (1.13–2.88)	0.013
Prior WBRT (No vs. Yes)	1.81 (0.67–4.92)	0.244
Salvage SRS to Same Lesion (No vs. Yes)	0.42 (0.10–1.72)	0.228
Prior RN at Other Lesions (No vs. Yes)	0.64 (0.34–1.20)	0.164
Number of SRS Targets	0.90 (0.83–0.96)	0.003
Prescription Dose (Gy)	0.93 (0.84–1.03)	0.183
Maximum Dose (Gy)	1.05 (1.02–1.07)	0.001
Maximum Lesion Dimension (cm)	1.31 (1.18–1.45)	< 0.0001
Target Volume (cc)	1.23 (1.15–1.31)	< 0.0001
Prescription Isodose Line (< 70% vs. ≥ 70%)	2.16 (1.49–3.14)	< 0.0001
Heterogeneity Index	3.37 (1.88–6.05)	< 0.0001
Conformality Index	0.45 (0.29–0.71)	0.001
Gradient Index	0.88 (0.82–0.95)	0.002
V10 (cc)	1.05 (1.03–1.06)	< 0.0001
V12 (cc)	1.06 (1.04–1.09)	< 0.0001

Higher maximum dose (*p* = 0.001) had a greater risk of RN, as did the receipt of concurrent targeted therapy (*p* = 0.034) or chemotherapy (*p* = 0.009). Receipt of immunotherapy trended towards but did not meet significance (*p* = 0.057).

Prescription isodose line of < 70% (*p* < 0.0001), greater V10 (*p* < 0.0001) and greater V12 (*p* < 0.0001), lower conformality index (*p* = 0.001), lower gradient index (*p* = 0.002), and higher heterogeneity index (*p* < 0.0001) were also associated with higher risk of RN.

Other factors, including age (*p* = 0.809), gender (*p* = 0.342), prior WBRT (*p* = 0.244), prior SRS to the same lesion (*p* = 0.228), prior RN at other treatment site (*p* = 0.164), and prescription dose (*p* = 0.183) were not significantly associated with risk of RN.

### Recursive partitioning analysis

Variables identified for inclusion in the RPA included primary tumor pathology, tumor location, maximum lesion diameter, maximum dose, prescription isodose line, lesion V10, lesion V12, heterogeneity index, number of SRS targets, prior SRS to other lesions, pre-SRS hemorrhage, post-SRS hemorrhage, targeted therapy within 2 weeks, immunotherapy within 2 weeks, and chemotherapy within 2 weeks. While significant on univariate analysis, target volume, conformality index, and gradient index were excluded from the RPA analysis, as they were determined to be closely dependent on other included variables—particularly maximum lesion diameter.

**Fig. 1:**
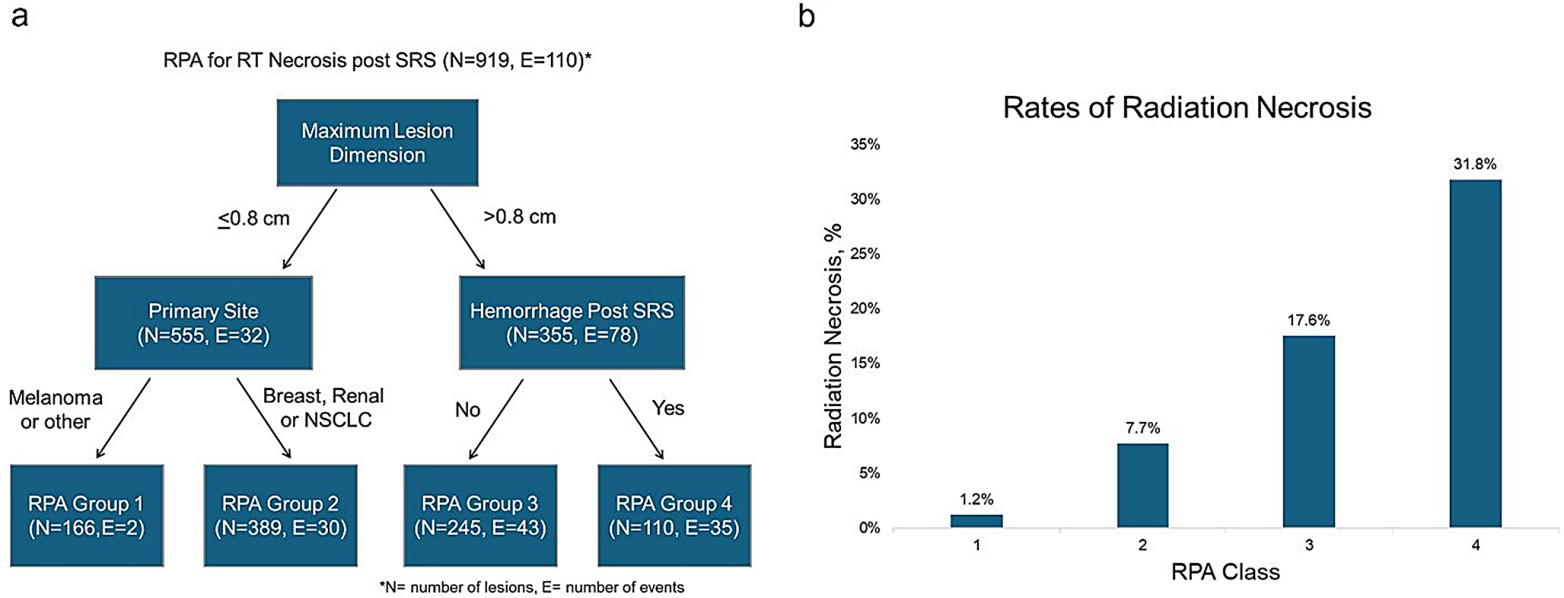
RPA tree for RN for patients with brain metastases treated with single fraction SRS (**a**) and rates of RN by RPA class (**b**)

**Fig. 2:**
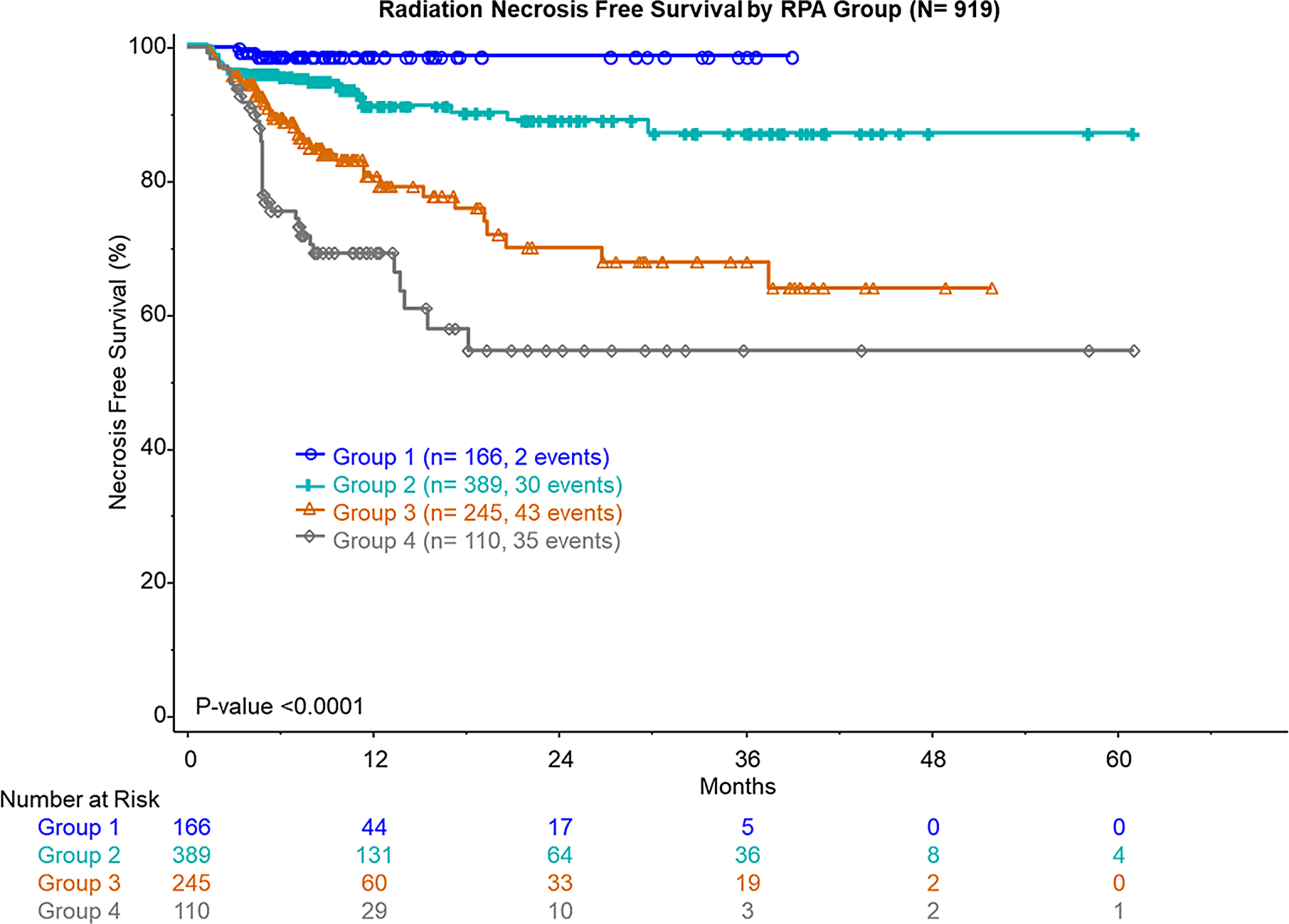
Kaplan Meier Curve for RN free survival by RPA class. Group 1: Lesion Maximum Dimension < 0.8 and Primary Site = Melanoma or Other. Group 2: Lesion Maximum Dimension < 0.8 and Primary Site = Breast, NSCLC, or Renal. Group 3: Lesion Maximum Dimension > 0.8 and Post SRS Hemorrhage = No. Group 4: Lesion Maximum Dimension > 0.8 and Post SRS Hemorrhage = Yes

RPA identified four distinct groups (Fig. 1a). Group 1 was maximum lesion diameter (MLD) ≤ 0.8 cm with primary tumor site other than breast, NSCLC, or renal (*n* = 166); group 2 was MLD ≤ 0.8 cm with primary tumor site of breast, NSCLC, or renal (*n* = 389); group 3 was MLD > 0.8 cm without post-SRS hemorrhage (*n* = 245); and group 4 was MLD > 0.8 cm with post-SRS hemorrhage (*n* = 110). The rate of RN in each group was 1.2%, 7.7%, 17.6%, and 31.8%, respectively (Fig. 1b). One-year RN free survival was 87% (95% CI: 84–89%) for all lesions, 99% (95% CI: 97–100%) for group 1, 91% (95% CI: 87–94%) for group 2, 80% (95% CI: 74–87%) for group 3, and 67% (95% CI: 57–77%) for group 4. Two-year RN free survival was 81% (95% CI: 77–85%) for all lesions, 99% (95% CI: 97–100%) for group 1, 89% (95% CI: 85–93%) for group 2, 70% (95% CI: 61–80%) for group 3, and 52% (95% CI: 38–66%) for group 4 (Fig. 2).

## Discussion

While SRS is associated with high rates of local control, the development of RN poses a potential challenge for managing post-treatment complications. This study presents the first RPA designed to stratify patients based on their risk of developing RN following single-fraction SRS. In this cohort of 170 patients with 919 brain metastases, the incidence of any RN was 12%, while the incidence of symptomatic RN was 3.5%, at a median of 4.9 months after SRS, consistent with previous reports that estimate the incidence of RN after SRS to be as high as 20% [1, 9, 11].

Univariate analysis revealed several significant predictors of RN involving lesion characteristics, including lesion size, location, and pathology, as well as dosimetric values, including maximum radiation dose, V10, V12, conformality index, gradient index, and heterogeneity index. These risk factors remain largely consistent with existing literature, including models to predict risk of injury after SRS for arteriovenous malformation [9, 11, 12, 15–18]. The identification of concurrent systemic therapies, particularly targeted therapy and chemotherapy, as significant risk factors underscores the potential for these treatments to exacerbate radiation effects, warranting careful consideration during treatment planning [7, 8, 13, 14, 19]. While immunotherapy has been previously established as a risk factor for symptomatic RN following SRS, the current analysis did not find a significant association [13]. However, this may be limited by sample size as receipt of immunotherapy showed a trend toward significant association on univariate analysis. Moreover, this analysis revealed the impact of post-SRS hemorrhage, with the presence of such hemorrhage dramatically increasing the risk of RN. To our knowledge, post-SRS hemorrhage has not been previously reported in literature as a risk factor for RN; however, this finding may be explained by the hypothesis that compromised vascular integrity after SRS may predispose patients to necrotic changes [20, 21].

The RPA yielded four distinct risk groups, providing a nuanced approach to predicting RN following single-fraction SRS. Notably, patients with MLD less than 0.8 cm and primary tumor sites other than breast, NSCLC, or renal demonstrated a minimal RN rate of 1.2%. In contrast, the group with MLD greater than 0.8 cm and post-SRS hemorrhage faced an alarming RN rate of 31.8%. Interestingly, while maximum dose was a significant predictor for RN on univariate analysis, it ultimately fell out of the RPA model, perhaps due to limited heterogeneity within the cohort. The observed one-year RN free survival rates further reinforce the clinical utility of this model, with rates of 99% and 91% for the low-risk groups compared to 67% in the high-risk cohort. Patients at higher risk based on pre-treatment tumor size and histology and development of post-SRS hemorrhage may warrant additional counseling and closer imaging follow-up.

Diagnosis of RN remains a challenge with conventional imaging techniques. On T1-weighted with contrast and T2/FLAIR sequences of an MRI, radiation necrosis may appear as a ring-enhancing lesion with surrounding vasogenic edema. However, these findings are non-specific and can also be seen in the setting of tumor recurrence or infection. There is an increasing use of perfusion MRI to measure relative cerebral blood volume (rCBV), which can be a surrogate for intact vasculature and neovascularization seen in the setting of viable tumor and less likely with RN [6]. Studies have suggested an rCBV of 1.5-2 to distinguish between RN and tumor recurrence [22, 23]. When using fluciclovine PET, maximum standardized uptake values of 1.3–4.3 have been shown to accurately differentiate RN from tumor progression [24, 25]. Other diagnostic tools that have been studied include FDG PET, amino acid PET, and MR spectroscopy [6].

The stratification from this RPA offers a valuable tool for personalized risk assessment and can potentially guide decision-making in the post-SRS setting. This data can be utilized when determining how much weight should be given to the accuracy of current and future imaging tools when deciding whether to proceed with medical management of RN vs. intervention for tumor progression. For instance, rCBV may have a sensitivity of 91% and specificity of 72% according to Barajas et al. [23]. If the rCBV is diminished on a lesion increasing in size, but RPA suggest a low pretest probability of necrosis, the diagnosis of necrosis based on imaging may come into question. Likewise, if the rCBV is increased, but RPA suggest a high pretest probability of necrosis, again the diagnosis of recurrence may come into question. Conversely if the pretest probability from the RPA for a given diagnosis is concordant with the imaging, it will provide reassurance that the result of the test is correct. Future studies looking at newer imaging techniques are being conducted, which will report various sensitivities and specificities of these modalities, but this RPA may help to refine the diagnostic accuracy of these tests [24].

The treatment of RN is often empiric, and can include observation, medical therapy, or invasive procedures. For small, asymptomatic lesions, observation with close interval follow-up may be reasonable. Oral steroids are generally considered first-line treatment for symptomatic RN. Other medical therapies include pentoxifylline/vitamin E or bevacuzimab [6, 26, 27]. Recently, Boswelia Serrata has been shown to be another potential treatment option for RN given its anti-inflammatory properties [28]. Other management options include invasive procedures such as resection, laser interstitial thermal therapy, and hyperbaric oxygen [6, 29–31].

This study has several important limitations. As a single-institution retrospective analysis, the findings may not be generalizable to broader populations due to potential selection bias for tumors considered optimal for single-fraction SRS. Moreover, the results may not apply to patients receiving fractionated SRS or those with larger metastases. The limited follow-up duration of 9 months could also lead to an underestimation of the actual incidence of RN, as RN could continue to develop several years after SRS. Long-term follow-up remains particularly important as systemic therapies continue to evolve, thus potentially limiting the utility of this model. Further validation of the RPA model with long-term follow-up as well as across multiple institutions is necessary to confirm its robustness. Additionally, incorporating advanced imaging modalities in future studies could enhance predictive capabilities, helping to better differentiate RN from tumor recurrence. Despite these limitations, the development of the RPA model provides valuable insights for identifying high-risk patients and improving clinical risk management strategies.

## Conclusion

This is the first RPA model for predicting the risk of RN following single-fraction SRS for brain metastases. By identifying patient and treatment specific risk factors, this model facilitates personalized treatment approaches and identifies high risk patients who may require appropriate counseling and close monitoring. Longer follow-up and multi-institutional validation are necessary to confirm the predictive value of the RPA.

## Data Availability

No datasets were generated or analysed during the current study.
